# Neuroprotective Effects of Biochanin A against β-Amyloid-Induced Neurotoxicity in PC12 Cells via a Mitochondrial-Dependent Apoptosis Pathway

**DOI:** 10.3390/molecules21050548

**Published:** 2016-04-25

**Authors:** Ji Wei Tan, Min Kyu Kim

**Affiliations:** 1 Department of Biomedical Sciences, Faculty of Medicine and Health Sciences, Universiti Putra Malaysia, 43400 Serdang, Selangor, Malaysia; jwtan1987@gmail.com; 2 Institute of Bioscience, Universiti Putra Malaysia, 43400 Serdang, Selangor, Malaysia

**Keywords:** biochanin A, beta-amyloid, apoptosis, PC12 cells, mitochondrial dysfunction, Alzheimer’s disease

## Abstract

Alzheimer’s disease is considered one of the major neurodegenerative diseases and is characterized by the production of β-amyloid (Aβ) proteins and progressive loss of neurons. Biochanin A, a phytoestrogen compound found mainly in *Trifolium pratense*, was used in the present study as a potential alternative to estrogen replacement therapy via the investigation of its neuroprotective effects against Aβ_25–35_-induced toxicity, as well as of its potential mechanisms of action in PC12 cells. Exposure of these cells to the Aβ_25–35_ protein significantly increased cell viability loss and apoptosis. However, the effects induced by Aβ_25–35_ were markedly reversed in the present of biochanin A. Pretreatment with biochanin A attenuated the cytotoxic effect of the Aβ_25–35_ protein by decreasing viability loss, LDH release, and caspase activity in cells. Moreover, we found that expression of cytochrome c and Puma were reduced, alongside with the restoration of Bcl-2/Bax and Bcl-xL/Bax ratio in the presence of biochanin A, which led to a decrease in the apoptotic rate. These data demonstrate that mitochondria are involved in the protective effect of biochanin A against Aβ_25–35_ and that this drug attenuated Aβ_25–35_-induced PC12 cell injury and apoptosis by preventing mitochondrial dysfunction. Thus, biochanin A might raise a possibility as a potential therapeutic agent for Alzheimer’s disease and other related neurodegenerative diseases.

## 1. Introduction

Alzheimer’s disease (AD), characterized by the development of amyloid plaques, neurofibrillary tangles, and neuronal cell death, is the most prevalent and age-related neurodegenerative disease among the elderly [1,2]. The β-amyloid (Aβ) protein is the major component of senile plaques and has a causal role in the development and progression of AD [3]. Its peptide fragment chain Aβ_25–35_ is the most toxic derivative, and has been universally applied in the research of AD [4]. Although the actual mechanism associated with Aβ-induced toxicity remains elusive, some studies have demonstrated evidence that oxidative stress and mitochondrial dysfunction play a role in Aβ-mediated neuronal cytotoxicity [5,6,7].

Mitochondrial dysfunction has been well researched to play a critical role in the early pathology of AD [8,9,10]. Several recent review studies have reported that extracellular Aβ causes oxidative stress to the mitochondria, which thus leads to its inevitable dysfunction [11,12]. Additionally, *in vivo* studies have also reported that Aβ is highly linked to mitochondrial structural abnormalities, as it was found to accumulate within mitochondria in the brains of AD patients [13,14]. Such an event leads to the release of cytochrome c as well as other apoptotic inducing proteins, from the mitochondria [15,16].

In recent years, noticeable efforts have been made to evaluate the beneficial effects of phytoestrogen on human health, including the prevention of cancers, heart disease and menopausal symptoms [17,18,19]. Biochanin A is an *O*-methylated isoflavone that is considered as a phytoestrogen compound, and is found mainly in legume plants, such as *Trifolium pratense* and soy [20]. Its chemical structure resembles that of endogenous estrogen, which is able to displays agonistic and antagonistic interactions with the estrogen receptor. Over the past few years, biochanin A has been applied in research on cancer prophylaxis and neuroprotection studies [20,21,22,23]. Despite the many considerable efforts to study the beneficial effects of biochanin A, there remains a significant lack of understanding of the effects of this compound on the central nervous system.

Our previous study showed that biochanin A attenuates glutamate-induced cytotoxicity in PC12 cells, which suggests that this phytoestrogen compound functions as a link between neuroprotection and the CNS [24]. Therefore, because of the possibility that phytoestrogen can become a frontier against neurodegenerative diseases, we investigated the neuroprotective effects of biochanin A on PC12 cells and its ability to attenuate the neurotoxin peptide of Aβ. In the current study, we discovered that biochanin A plays a vital part in the inhibition of Aβ-induced cytotoxicity.

## 2. Results

### 2.1. Protective Effect of Biochanin A against Aβ_25–35_-Induced Cytotoxicity

**Figure 1:**
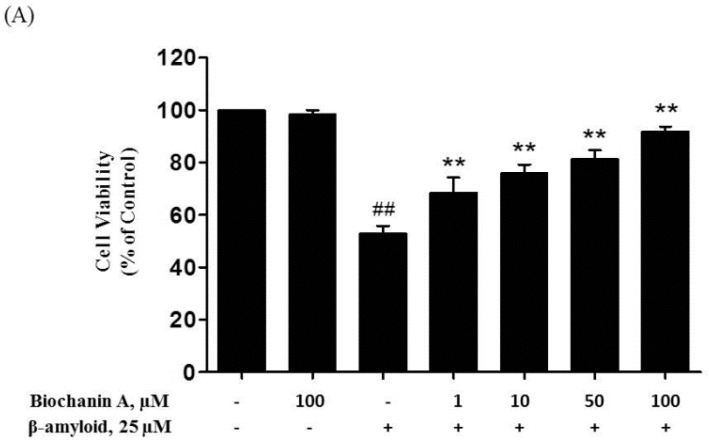

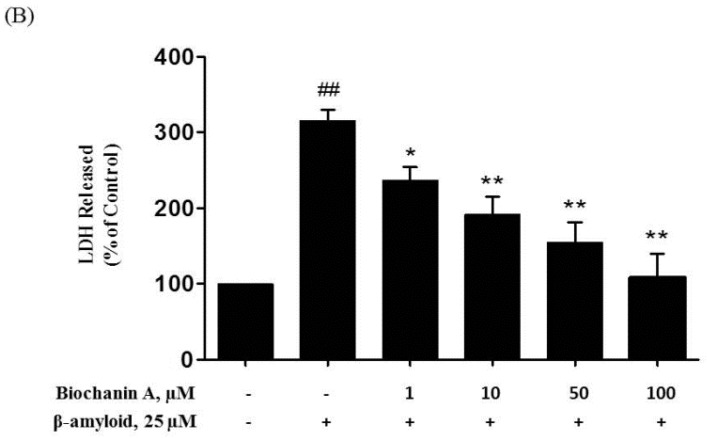
Effect of biochanin A on Aβ_25–35_-induced cytotoxicity and LDH leakage. (**A**) Cell viability as assessed using the MTT assay. PC12 cells were exposed to 25 μM Aβ_25–35_ for 24 h in the presence or absence (control) of biochanin A (1, 10, 50, and 100 μM); (**B**) The release of LDH into extracellular surroundings was measured using the LDH assay. PC12 cells were either untreated (control) or treated with 25 μM Aβ_25–35_ with or without biochanin A for 24 h. Each value represents the mean ± S.E.M. from four independent experiments. * *p* < 0.05 and ** *p* < 0.01 compared with the group treated with Aβ_25–35_ alone; ^##^ *p* < 0.01 compared with the control group. “−”and “+”in the figure indicate “in the absent of” and “in the present of” respectively.

The MTT test of cell viability showed that biochanin A attenuated the cytotoxic effect of Aβ_25–35_ in a dose-dependent manner up to 100 μM (Figure 1). Apparently, 100 μM biochanin A alone did not cause any harm in PC12 cells. Treatment of PC12 cells with 25 μM Aβ_25–35_ alone led to a significant decrease in cell viability to 53.25% ± 1.74% compared with the control group. However, pretreatment with biochanin A (1–100 μM) reversed the Aβ_25–35_-induced cell death in a concentration-dependent manner (EC_50_ of 24.3 μM).

Next, we determined the level of LDH release, which is also an indicator of cell injury, from the treated PC12 cells. The colorimetric assay revealed that exposure to Aβ_25–35_ alone induced a significant increase in LDH release into the medium, by 315.82% ± 14.16% (Figure 1) compared with the control group. Conversely, in the presence of biochanin A, the effect of Aβ_25–35_ on LDH release was reduced compared with that in the case of the control group (IC_50_ of 19.5 μM).

### 2.2. Effect of Biochanin A on Suppressing Cell Apoptosis Induced by Aβ_25–35_

**Figure 2:**
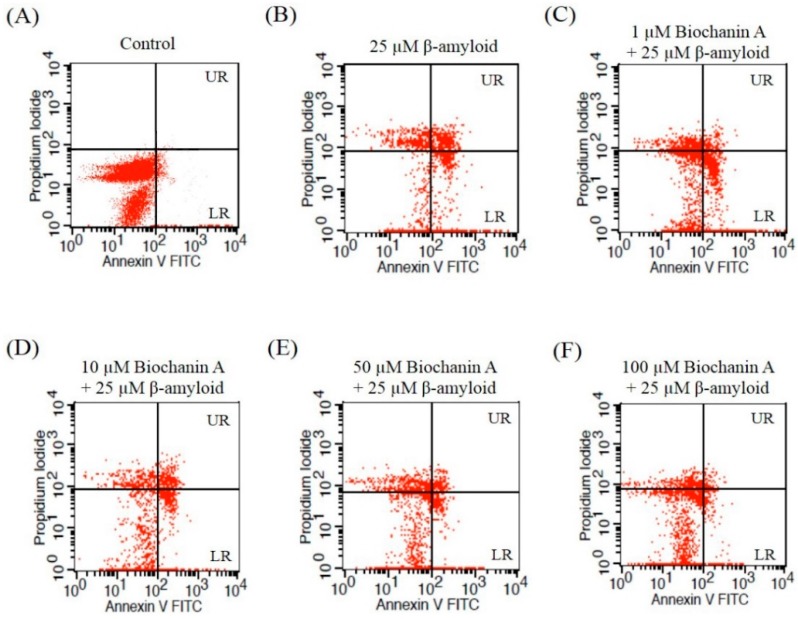

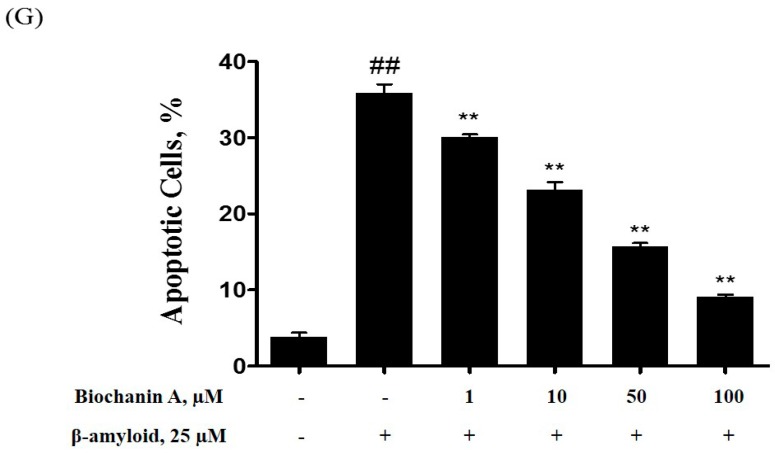
Effect of biochanin A on Aβ_25–35_-induced apoptosis as measured by flow cytometry. After PC12 cells were plated, they were either unexposed (control) or exposed to 25 μM Aβ_25–35_ with or without biochanin A (1, 10, 50, and 100 μM). After 24 h of treatment, the treated PC12 cells were harvested and labeled with annexin V–FITC and PI and further analyzed by flow cytometry. The figure shows representative flow cytometric quadrants and histogram. (**A**) Control; (**B**) 25 μM Aβ_25–35_ alone; (**C**) 1 μM biochanin A + 25 μM Aβ_25–35_; (**D**) 10 μM biochanin A + 25 μM Aβ_25–35_; (**E**) 50 μM biochanin A + 25 μM Aβ_25–35_; (**F**) 100 μM biochanin A + 25 μM Aβ_25–35_; (**G**) The number of count in the UR and LR region of the flow cytometry quadrant were counted as apoptotic cells. Each value represents the mean ± S.E.M. relative to the control from four independent experiments. ** *p* < 0.01 compared with the group treated with Aβ_25–35_ alone; ^##^ *p* < 0.01 compared with the control group. “−”and “+”in the figure indicate “in the absent of” and “in the present of” respectively.

Apoptotic cell death plays a vital role in the pathogenesis of AD. To examine whether the cell death induced by Aβ_25–35_ is apoptosis like, flow cytometry analysis using annexin V and PI double staining was performed. As shown in Figure 2, exposure to 25 μM Aβ_25–35_ alone significantly increased early (LR) and late (UR) apoptotic cell death in PC12 cells, with a total rise in the number of apoptotic cells of up to 35.82% ± 1.18%. However, preincubation with biochanin A resulted in a significant decrease in the apoptotic rate (IC_50_ of 19.2 μM).

**Figure 3:**
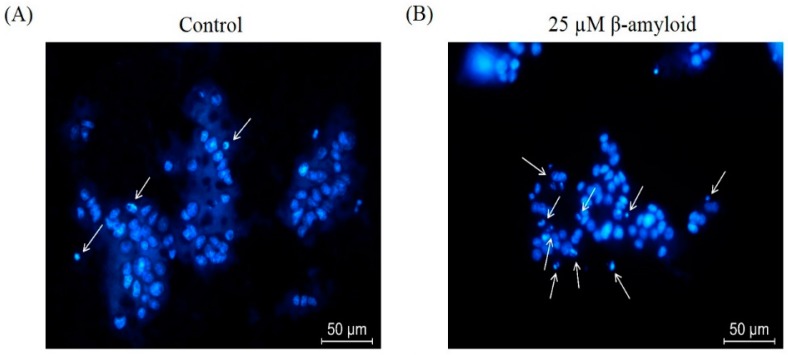

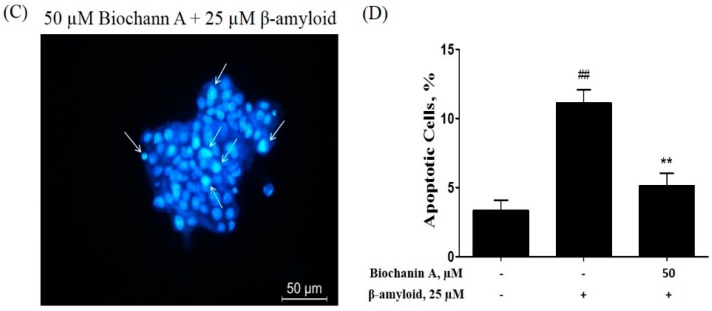
Effect of biochanin A on Aβ_25–35_-induced nuclear condensation. PC12 cells were treated with Aβ_25–35_ (25 μM) for 24 h in the presence or absence of biochanin A (50 μM). The cells were observed by fluorescence microscopy after nuclei were stained with Hoechst 33342. (**A**) Control cells; (**B**) Cells treated with Aβ alone; (**C**) Cells treated with Aβ_25–35_ and biochanin A (50 μM). Arrows indicating the nuclei condensation and apoptotic bodies; (**D**) Five randomized representative fields from each group were analyzed in one experiment. Each value represents the mean ± S.E.M. relative to the control from four independent experiments. ** *p* < 0.01 compared with the group treated with Aβ_25–35_ alone; ^##^ *p* < 0.01 compared with the control group. “−”and “+”in the figure indicate “in the absent of” and “in the present of” respectively.

In addition, we performed Hoechst 33342 staining to observe the nuclear morphological changes associated with apoptosis. In Figure 3, compared with the non-treated control group, PC12 cells treated with Aβ_25–35_ alone exhibited typical apoptosis characteristics, such as highly condensed and fragmented nuclei, when observed under a fluorescence microscope. However, biochanin A prevented these manifestations during exposure to Aβ_25–35_, resulting in the detection of more round and homogeneously stained nuclei, similar to those observed in control cells.

### 2.3. Biochanin A Suppressed Aβ_25–35_-Induced Caspase Activity

**Figure 4:**
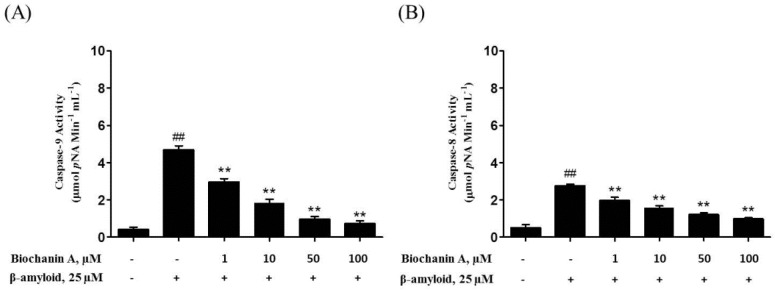

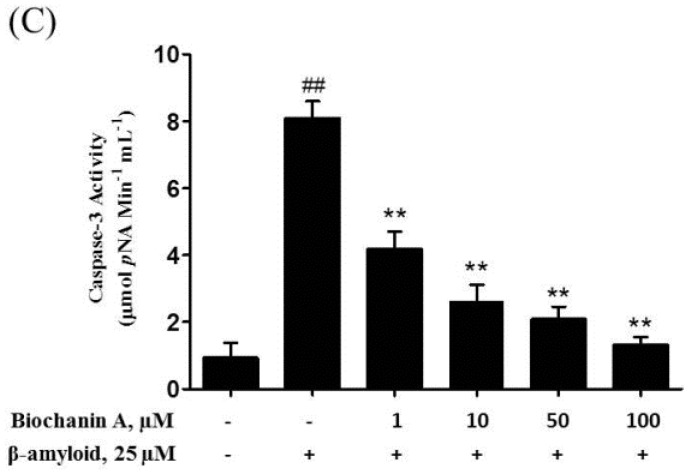
Effect of biochanin A on Aβ_25–35_-induced caspase activity. PC12 Cells were plated on a collagen type I-coated 6-well plate for 24 h. Subsequently, they were either treated or not (control) with 25 μM of Aβ_25–35_ with or without biochanin A (1, 10, 50, and 100 μM). Treated PC12 cells were harvested and subjected to (**A**) caspase-9; (**B**) caspase-8; and (**C**) caspase-3 activity tests. All data represent the mean ± S.E.M. of four independent experiments. ** *p* < 0.01 compared with the group treated with Aβ_25–35_ alone; ^##^ *p* < 0.01 compared with the control group. “−”and “+”in the figure indicate “in the absent of” and “in the present of” respectively.

Apoptotic cell death involves the activation of various caspases, which play a main role in activating and regulating the whole apoptosis process. After 24 h of treatment of PC12 cells with 25 μM Aβ_25–35_, an increase in the activity of caspase-3 (8.10 ± 0.50 μmol pNA min^−1^ mL^−1^), caspase-8 (2.77 ± 0.09 μmol pNA min^−1^ mL^−1^), and caspase-9 (4.70 ± 0.21 μmol pNA min^−1^ mL^−1^) was detected (Figure 4). In contrast, PC12 cells that were pretreated with biochanin A exhibited a significant decrease in the activity of all 3 caspases compared with cells treated with Aβ_25–35_ alone at the same time point (IC_50_ = 20.6 μM, 24.2 μM, and 19.9 μM respectively).

### 2.4. Effect of Biochanin A on Aβ_25–35_-Induced MMP Collapse

**Figure 5:**
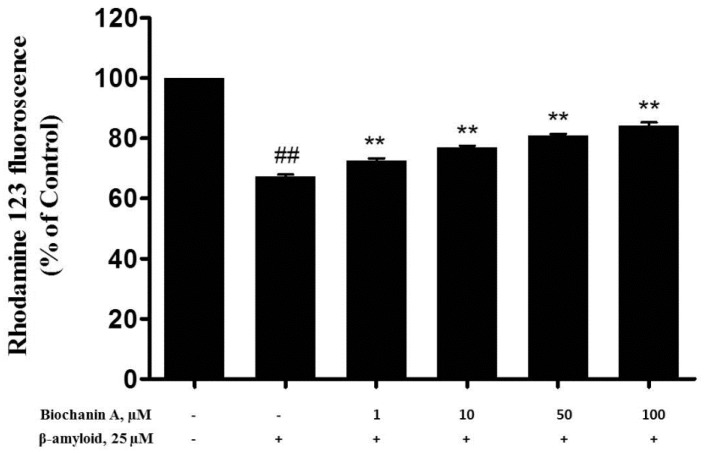
Effect of biochanin A on the mitochondrial membrane potential in Aβ_25–35_-treated PC12 cells. The cells were pretreated with biochanin A (1, 10, 50, and 100 μM) for 2 h before exposure to 25 μM Aβ_25–35_ for another 24 h. Values given are the mean ± S.E.M. of four independent experiments. ** *p* < 0.01 compared with the group treated with Aβ_25–35_ alone; ^##^ *p* < 0.01 compared with the control group. “−”and “+”in the figure indicate “in the absent of” and “in the present of” respectively.

According to a previous report, the depolarization of MMP results in the loss of Rh123 from mitochondria, causing a decrease in the intracellular fluorescence [25]. Therefore, to characterize the changes in mitochondrial events induced by Aβ_25–35_ and/or biochanin A treatment, the collapse of MMP in PC12 cells was monitored using the rhodamine 123 probe. As shown in Figure 5, our results indicated that rhodamine 123 fluorescence intensity was significantly reduced (67.35% ± 0.72%) after incubating the cells with 25 μM of Aβ_25–35_ for 24 h. However, pretreatment with biochanin A led to a significant increase in the fluorescent intensities in a dose-dependent manner (EC_50_ = 16.9 μM).

### 2.5. Effect of Biochanin A on the Expression of Pro- and Antiapoptotic Proteins in PC12 Cells Treated with Aβ_25–35_

**Figure 6:**
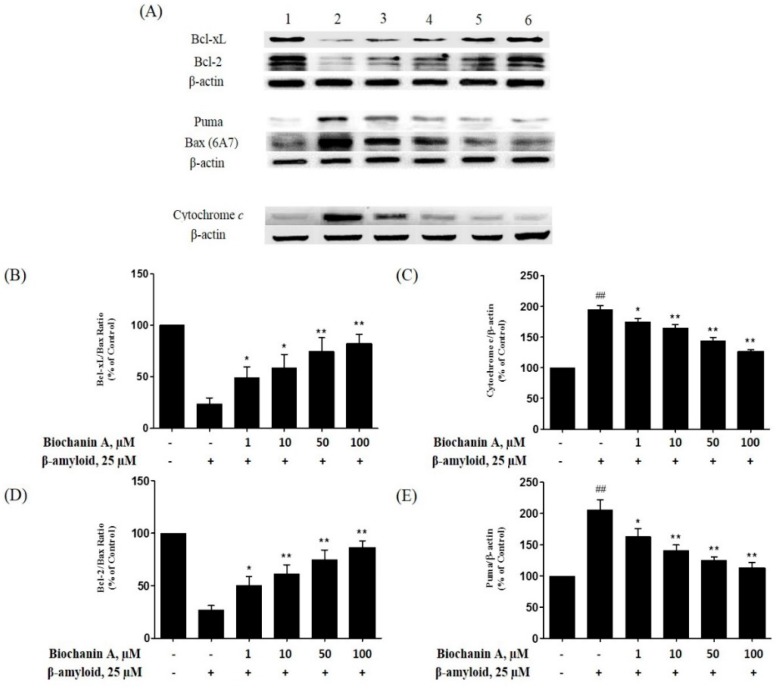
Effect of biochanin A on the expression of Bcl-2, Bcl-xL, Bax, Puma and cytochrome c in treated PC12 cells. Cells were plated for 24 h in culture medium; subsequently, they were either exposed or not (control) to 25 μM Aβ_25–35_ in the presence or absence of biochanin A (1, 10, 50, and 100 μM). The western blotting technique was used to detect the expression of (**A**) Bcl-2, Bcl-xL, Bax, Puma and cytochrome c on a PVDF membrane. The intensity of the expression was determined using the ImageJ program and plotted for (**B**) Bcl-xL/Bax ratio; (**C**) cytochrome c; (**D**) Bcl-2/Bax ratio; and (**E**) Puma. (**1**) Control; (**2**) 25 μM Aβ_25–35_ alone; (**3**) 1 μM biochanin A + 25 μM Aβ_25–35_; (**4**) 10 μM biochanin A + 25 μM Aβ_25–35_; (**5**) 50 μM biochanin A + 25 μM Aβ_25–35_; (**6**) 100 μM biochanin A + 25 μM Aβ_25–35_. Each value represents the mean ± S.E.M. of 4 independent experiments. * *p* < 0.05 and ** *p* < 0.01 compared with the Aβ_25–35_-treated group; ^##^ *p* < 0.01 compared with the control group. “−”and “+”in the figure indicate “in the absent of” and “in the present of” respectively.

The ratio between Bcl-2/Bcl-xL and Bax has been previously shown to correlate well with apoptosis [26]. Thus, to investigate the molecular mechanisms underlying the protective effect of biochanin A against Aβ_25–35_-induced cell apoptosis, we examined the expression of Bcl-2, Bcl-xL, Bax, Puma and cytochrome c. As shown in Figure 6B,D, Aβ_25–35_-treated cells showed a decrease in both Bcl-xL/Bax and Bcl-2/Bax expression ratio (27.23% ± 3.86% and 23.83% ± 4.95% respectively). However, pretreatment with biochanin A prevented the Aβ_25–35_-induced downregulation of both anti-apoptotic proteins and upregulation of Bax, resulting in an increase in both expression ratio. In the case of cytochrome c and Puma, Aβ_25–35_ causes approximately 2-fold increase in their expression (194.54% ± 7.17% and 206.07% ± 13.54% respectively) compared with the control group. Conversely, as shown in Figure 6C,E, their expression was decreased in a dose-dependent manner in the presence of biochanin A.

## 3. Discussion

Phytoestrogens are plant-based molecules that structurally resemble endogenous estrogen. They can bind easily to estrogen receptors (ERs) to regulate gene expression, which allows them to exert their estrogenic and/or antiestrogenic effects [27]. Over the past few years, phytoestrogens have received much attention as a potential alternative to estrogen therapy [28]. They have also been suggested as novel candidate drugs for AD [29]. As one of the phytoestrogen compounds, biochanin A, has been shown to have an important effect in cancer protection [21,22]. However, there is still much to learn about this phytoestrogen and its beneficial effects on human health. Thus, in the present study, we evaluated the neuroprotective mechanism(s) of biochanin A against Aβ-induced cytotoxicity. The results of our study showed that biochanin A attenuated Aβ_25–35_-induced PC12 cell cytoxicity and apoptosis, which involved the modulation of the expression of pro- and antiapoptotic proteins, specifically Bcl-2, Bcl-xL, Bax, Puma and cytochrome c.

Aβ accumulation has been linked often to neuronal dysfunction and neuronal loss during the pathogenesis of AD [30]. In this study, we first examined whether biochanin A could protect PC12 cells from Aβ_25–35_-induced toxicity. Our results showed that 25 μM Aβ_25–35_ significantly reduced cell viability. However, preincubation with biochanin A attenuated the cytotoxic effect of Aβ_25–35_, where a higher percentage of cell viability was detected in the MTT assay. Moreover, the protective effect of biochanin A was dose dependent, with a maximum effect observed in the group that was pretreated with 100 μM biochanin A. To justify the protective effect of biochanin A on Aβ_25–35_-induced toxicity, an LDH assay was performed. LDH is a stable cytoplasmic enzyme that can be found in all cells. It is also another indicator of cell toxicity and is rapidly released into the extracellular milieu when the plasma membrane is damaged [31]. Therefore, LDH is used frequently as a reliable indicator of neuronal plasma membrane damage [32]. The results of our study indicated that Aβ_25–35_-treated cells exhibited a significant increase in LDH release, whereas the cells pretreated with biochanin A released less LDH.

Aβ insults cause neuronal cell death via the activation of apoptosis [31,33]. Consistent with previous findings that have been reported by other researchers, our quantification results of annexin V and PI double staining showed that Aβ_25–35_ significantly increased the number of apoptotic cells detected and that its effect was attenuated in the presence of biochanin A. Moreover, to confirm our results, we examined the nuclear morphological changes associated with apoptosis via Hoechst 33342 staining. Cells treated solely with 25 μM Aβ_25–35_ exhibited typical characteristics of apoptosis, such as highly condensed nuclei and cell shrinkage.

Given that biochanin A attenuated the effect of Aβ_25–35_-induced apoptosis, it is not surprising that biochanin A treatment may also be associated with the inhibition of the downstream apoptotic signaling pathways. Caspases (cysteine-requiring aspartate proteases) are involved in regulating these downstream signaling pathways [34]. It has also been suggested that caspases play a role in executing the apoptotic process [34]. They act via two pathways: the death receptor pathway (extrinsic) and the mitochondrial pathway (intrinsic). Each of the different pathways requires different caspases to activate, although the apoptotic signal will ultimately converge into the main executor, which is caspase-3 [34]. In our study, we found that Aβ_25–35_ significantly increased both caspase-8 and caspase-9 activity, which eventually led to the increase in caspase-3 activity. Conversely, the activity of all 3 caspases decreased after pretreatment with biochanin A.

As shown here, the activities of both caspase-8 and caspase-9 significantly increased after treatment of PC12 cells with Aβ_25–35_. Interestingly, we noticed that the increase in caspase-9 activity was greater than that was observed for caspase-8 and pretreatment with biochanin A inhibited the activity of caspase-9 more effectively than it did in caspase-8. As such, we believe that biochanin A exerts its effect mainly via the inhibition of the intrinsic apoptotic pathway. Therefore, we tried to investigate the role of biochanin A in Aβ_25–35_-induced mitochondrial dysfunction.

Mitochondria have been reported often to play an important role in the regulation of cell death, particularly cell apoptosis [35]. As mitochondrial dysfunction is a hallmark of Aβ-induced neuronal toxicity in AD [8,9,10], there is no doubt that the Bcl-2-family proteins are involved in the regulation of neuronal apoptotic cell death [36,37,38]. To date, the exact mechanism which the Bcl-2 family proteins act upon the apoptotic pathway is still not fully understood. Nevertheless, the ratio between the pro- and anti-apoptotic proteins is one of the critical factors that determine the apoptotic state of cells, rather than just the absolute concentration of either molecule alone [35].

According to a previous reported studies, the Bcl-2 family proteins regulate apoptosis by modulating mitochondrial permeability [39,40]. There is also a previous report which shows that Aβ causes the collapse of the MMP, resulting in the opening of mitochondrial permeability transition (MPT) pores [40]. This event leads to the release of certain important proteins, such as cytochrome c or other apoptosis-inducing factors, into the cytosol [15,41]. This release will eventually trigger the downstream part of the apoptotic cascade [42]. Our experimental results regarding the ability of Aβ to decrease MMP are consistent with previous findings, where we showed that biochanin A effectively attenuated this adverse effect, as seen in Figure 5. Furthermore, our western blotting results showed that Aβ_25–35_ significantly decreased both Bcl-2/Bax and Bcl-xL/Bax expression ratio and, at the same time, upregulated cytochrome c in cells. It also increases the expression of Puma, the pro-apoptotic BH3-only proteins that responsible to activate Bax [43]. However, pre-exposure to biochanin A caused the occurrence of the completely reverse event, whereby in the presence of biochanin A, both Bcl-2/Bax and Bcl-xL/Bax expression ratio increases. At the same time, cytochrome c and Puma was downregulated.

Based on the western blotting observation of the pro- and anti-apoptotic proteins’ ratio in the apoptotic cascade, we showed that the decrease in caspase-3 activity correlated well with upstream signaling in the apoptotic pathway where it involves the restoration of MMP and increase in the Bcl-2/Bax and Bcl-xL/Bax ratio. Thus, it is suggested that the mechanism which biochanin A exerts its protective effects against Aβ_25–35_-induced caspase dependent apoptosis actually involves regulation by the Bcl-2 family proteins.

In summary, we demonstrated for the first time that biochanin A exerts an inhibitory effect on the adverse effects in Aβ_25–35_-treated PC12 cells. Based on the findings, we hypothesized that biochanin A exerts its neuroprotective effects by regulating the activity of the Bcl-2 family proteins prior to the activation of mitochondria-mediated downstream molecular events, such as cytochrome c release and caspase cascade activation. It is also possible that biochanin A has a modulating effect on the enzymes involved in other pathways that indirectly attenuate the Aβ-induced toxicity. However, before any definite conclusions can be drawn, further research is needed to determine all possible neuroprotective mechanisms of biochanin A.

## 4. Materials and Methods

### 4.1. Materials

PC12 cells (CRL-1721) were purchased from ATCC (Manassas, VA, USA). Dulbecco’s modified Eagle medium (DMEM) was purchased from Hyclone Laboratories (Logan, UT, USA). Fetal bovine serum, penicillin, and streptomycin were purchased from i-DNA Biotechnology (Singapore). The amyloid-β protein fragment 25–35 (Aβ_25–35_) was purchased from Bachem (Bubendorf, Switzerland). Biochanin A (MW: 284.26), rhodamine 123, and Hoechst 33342 (purity ≥ 98% by HPLC and TLC) were purchased from Sigma Aldrich (Saint Louis, MO, USA).

3-(4,5-Dimethylthiazol-2-yl)-2,5-diphenyltetrazolium bromide (MTT) and dimethyl sulfoxide (DMSO) were purchased from Amresco (Solon, OH, USA). The lactate dehydrogenase (LDH) assay kit and the caspase-3, caspase-8, and caspase-9 activity assay kit were purchased from BioVision (Milpitas, CA, USA). The FITC Annexin V Apoptosis Detection Kit II was purchased from Becton, Dickinson and Company, BD (Franklin Lakes, NJ, USA). Primary antibodies against cytochrome c, Bax, Bcl-2, and beta-actin, as well as their corresponding secondary antibodies, were purchased from Santa Cruz Biotechnology (Santa Cruz, CA, USA). Primary antibodies against Bcl-xL and Puma were purchased from Cell Signaling Technology (Danvers, MA, USA). Polyvinylidene difluoride (PVDF) membranes were purchased from Merck Millipore (Billerica, MA, USA). The Enhanced Chemiluminescence (ECL) reagent detection kit was purchased from Thermo Scientific Pierce (Rockford, IL, USA). The bicinchoninic acid protein assay kit was purchased from Aidlab Biotechnologies (Beijing, China).

### 4.2. Peptides

The Aβ_25–35_ peptides were dissolved in deionized water at 1 M as stock solution and aliquoted into smaller portions to keep them frozen until further use. To prepare the aggregated form of Aβ_25–35_, a solution of 1 mM was prepared in DMEM from the stock solution and incubated in a capped vial at 37 °C for 7 days. The desired concentration of 25 μM was prepared immediately prior to its use in experiments.

### 4.3. Cell Culture

PC12 cells were cultured in Dulbecco’s Modified Eagle’s Medium supplemented with 10% (*v*/*v*) of heat-inactivated fetal bovine serum (FBS) and 1% (*v*/*v*) of penicillin and streptomycin, followed by culture at 37 °C in a humidified atmosphere with 5% CO_2_. The cells were taken to be used in various experiments or were passaged once they reached 80% confluence. Prior to experiments, PC12 cells were seeded on collagen type I precoated plates at the desired cell density according to each experimental scale.

### 4.4. Cell Viability Assay

Cell viability was determined using the MTT reduction assay. Briefly, PC12 cells were seeded at 1 × 10^5^ cells/mL in 96-multiwell plates (precoated with collagen type I) to allow the cells to adhere on the wells of the plate. After 24 h of incubation, the cells were preincubated with biochanin A at different concentrations (0, 1, 10, 50, and 100 μM) for 2 h; subsequently, Aβ_25–35_ (final concentration of 25 μM) was added into each well, with the exception of the control group. The treated 96-multiwell plates were incubated for 24 h at 37 °C in an incubator. After the incubation period, MTT (20 μL, 5 mg/mL) was added to the treated cells, which were incubated for an additional 3 h at 37 °C. Finally, the media were carefully removed and formazan crystals were dissolved in 100 μL of DMSO. Absorbance was read at 570 nm using a microplate reader (Asys UVM 340, Biochrom, Cambourne, Cambridge, UK). Cell viability was expressed as the percentage of viable cells in the treated groups compared with that observed in the untreated control group.

### 4.5. Lactate Dehydrogenase Release Assay

Cytotoxicity was assessed quantitatively by measuring the lactate dehydrogenase (LDH) released from the damaged cells into the culture medium. PC12 cells were seeded into a 96-well culture plate (precoated with collagen type I) at a density of 2 × 10^5^ cell/mL. The seeded cells were then subjected to various treatments, as described in the MTT cell viability assay in Section 4.4. The measurement of LDH activity was performed following the protocols provided by the manufacturer of the assay kit. Briefly, the media from the treated PC12 cells were collected and centrifuged at 10,000× *g*. The supernatant (50 μL) was then mixed with an equal volume of reaction mixture to initiate the LDH reaction. The absorbance of each sample was read at 450 nm on a microplate reader. The data were normalized to the LDH release activity of the control cells.

### 4.6. Apoptosis Analysis

Apoptotic cells were quantified using the annexin V–FITC and PI apoptosis detection kit, according to the manufacturer’s instructions. Briefly, PC12 cells were harvested and washed twice with ice-cold phosphate-buffered saline (PBS) after treatment. The cells were then centrifuged at 800× *g* for 10 min, the supernatant was discarded, and the pellet was resuspended in binding buffer at a density of 1 × 10^6^ cells/mL. The suspension (100 μL) was transferred to a labeled tube and incubated with 5 μL of FITC-conjugated annexin V and 5 μL of PI for 15 min at room temperature in the dark. Then, 400 μL of binding buffer were added to each sample tube and analyzed by flow cytometry (FACSCalibur™, BD, Franklin Lakes, NJ, USA) within 1 h. Apoptotic cells were expressed as a percentage of the total number of cells.

### 4.7. Hoechst 33342 Staining Assay

The characteristic features of apoptotic nuclei were assessed using the Hoechst 33342 fluorescent dye. Briefly, PC12 cells were fixed with 4% paraformaldehyde and then stained with 5 mg/mL of Hoechst 33342 for 10 min. Nuclear morphology was visualized under a fluorescence microscope (Leica, Allendale, NJ, USA) to check for any sign of nuclear condensation or reduced nuclear size.

### 4.8. Caspase Activity Measurement

Caspase activity was assayed using the caspase-8, caspase-9, and caspase-3 activity assay kit, according to the manufacturer’s instructions. Briefly, PC12 cells were plated on 6-well culture plates at a density of 1 × 10^6^ cells/mL and treated with Aβ_25–35_ alone or accompanied by pretreatment with biochanin A for 2 h. After treatment, cells were collected and centrifuged at 1500 rpm for 10 min. Subsequently, the cells were resuspended in chilled cell lysis buffer for 10 min. The lysates were centrifuged for 5 min at 10,000× *g* and the supernatants were collected. Cell lysates (50 μL) were mixed with reaction buffer containing 200 μM of each substrate (DEVD-pNA for caspase-3 activity, LEHD-pNA substrate for caspase-9 activity, and IETD-pNA substrate for caspase-8 activity). After incubation for 1 h at 37 °C, absorbance in each well was measured at 405 nm on a microplate reader.

### 4.9. Mitochondrial Membrane Potential (MMP) Measurement

The MMP was measured using the rhodamine 123 fluorescent dye (Rh123), which is a cell-permeable cationic dye that preferentially partitions into mitochondria as a result of their highly negative MMP. Briefly, PC12 cells were seeded in 96-well black culture plates (precoated with collagen type I) at a density of 2 × 10^4^ cells/well. At the end of the drug treatment, the cells were washed with PBS and incubated with 10 μM rhodamine 123 for 30 min at 37 °C in the dark. After incubation, the cells were washed with PBS 3 times, and the fluorescence intensity was measured at an excitation wavelength of 488 nm and an emission wavelength of 510 nm using a fluorescence plate reader. The MMP was expressed as a percentage of that of the nontreated control.

### 4.10. Western Blot Analysis

Western blot analysis was performed to detect Bcl-2, Bcl-xL, Bax, Puma and cytochrome c. After exposure to Aβ_25–35_ and/or biochanin A for the indicated dosages and times, the cells were washed twice with PBS and treated with RIPA lysis buffer (50 mM Tris–HCl (pH 8.0), 150 mM NaCl, 1% Triton X-100, 0.5% sodium deoxycholate, and 0.1% SDS) containing a cocktail of protease inhibitors (2 μg/mL aprotinin, 1 mM phenylmethyl sulfonylfluoride, and 10 μg/mL leupeptin). The lysates were incubated for 30 min at 4 °C, centrifuged at 13,000× *g* for 15 min, and the protein concentrations were determined using a BCA protein assay kit. The cell lysates were electrophoresed on 12% SDS polyacrylamide gels and transferred to polyvinylidene difluoride (PVDF) membranes. The membranes were blocked in 5% BSA for 1 h at 4 °C and incubated with primary antibodies against Bcl-2, Bcl-xL, Bax, Puma, cytochrome c, and β-actin proteins overnight at 4 °C. The membranes were then incubated with their respective horseradish peroxidase-conjugated antibodies (anti-mouse IgG, anti-rabbit IgG, or anti-goat IgG) for 1 h at room temperature. The chemiluminescence signal of bonded antibodies was detected using the ECL reagent detection kit and was captured using a charge-coupled device (CCD) camera. The relative density of the protein bands was quantified by densitometry using the ImageJ program.

### 4.11. Statistical Analysis

The data were expressed as means ± S.E.M. values and evaluated using one-way ANOVA followed by Fisher’s Least Significant Difference (LSD) test as a post-hoc test. Significance was set at *p* < 0.05.
